# Thermal and non-thermal effects of capacitive–resistive electric transfer application on different structures of the knee: a cadaveric study

**DOI:** 10.1038/s41598-020-78612-8

**Published:** 2020-12-18

**Authors:** Jacobo Rodríguez-Sanz, Albert Pérez-Bellmunt, Carlos López-de-Celis, Orosia María Lucha-López, Vanessa González-Rueda, José Miguel Tricás-Moreno, Mathias Simon, César Hidalgo-García

**Affiliations:** 1 Faculty of Medicine and Health Sciences, Universitat Internacional de Catalunya, Barcelona, Spain; 2 ACTIUM Functional Anatomy Group, Campus Sant Cugat, c/Josep Trueta s/n, 08195 Sant Cugat del Vallès, Barcelona Spain; 3 Fundació Institut Universitari per a la recerca a l’Atenció Primària de Salut Jordi Gol i Gurina, Barcelona, Spain; 4 Facultad de Ciencias de la Salud, Universidad de Zaragoza, C/Domingo Miral S/N, 50009 Zaragoza, Zaragoza Spain; 5 Unidad de Investigación en Fisioterapia, Universidad de Zaragoza, C/Domingo Miral S/N, 50009 Zaragoza, Zaragoza Spain

**Keywords:** Anatomy, Diseases, Health care, Medical research

## Abstract

Capacitive–resistive electric transfer therapy is used in physical rehabilitation and sports medicine to treat muscle, bone, ligament and tendon injuries. The purpose is to analyze the temperature change and transmission of electric current in superficial and deep knee tissues when applying different protocols of capacitive–resistive electric transfer therapy. Five fresh frozen cadavers (10 legs) were included in this study. Four interventions (high/low power) were performed for 5 min by a physiotherapist with experience. Dynamic movements were performed to the posterior region of the knee. Capsular, intra-articular and superficial temperature were recorded at 1-min intervals and 5 min after the treatment, using thermocouples placed with ultrasound guidance. The low-power protocols had only slight capsular and intra-capsular thermal effects, but electric current flow was observed. The high-power protocols achieved a greater increase in capsular and intra-articular temperature and a greater current flow than the low-power protocols. The information obtained in this in vitro study could serve as basic science data to hypothesize capsular and intra-articular knee recovery in living subjects. The current flow without increasing the temperature in inflammatory processes and increasing the temperature of the tissues in chronic processes with capacitive–resistive electric transfer therapy could be useful for real patients.

## Introduction

The knee is one of the most frequently injured joints in physically active individuals^1–4^. Injury severity can range from asymptomatic injuries to damaged ligaments or menisci^5,6^. In the USA, anterior cruciate ligament injuries (ACL) are reported to occur in 250,000 individuals per year, with over 127,000 arthroscopic ACL reconstructions (ACLR) performed annually. ACL injuries are often not isolated: 43–70% of those undergoing ACLR have meniscal lesions, 20–25% have cartilage lesions (about 5% full-thickness) and over 80% have bone bruises^4,6–10^.

Many post-surgical rehabilitation guidelines are based on time from surgery and permit individuals to return to sports-specific activities after 4–9 months^11^. However, they don´t take into account joint junction. Knee pain, joint swelling, stiffness, instability, weakness, and joint effusion, are common reasons many athletes cite for not returning to preinjury activity levels^12–14^. All these pathologies can alter knee motion^4,15,16^.

Immobilization or limitation of range of motion (ROM) due to pain can induce joint contracture. This contracture may be influenced by two anatomical components around the joint: arthrogenic and myogenic components. Arthrogenic components, particularly of the joint capsule, are reported to be important factors in the formation of joint contractures. Previous studies have suggested that joint capsule fibrosis and overexpression of type I collagen occur and progress within 1 week after immobilization, and an increase in myofibroblasts is associated with this fibrosis^17^, especially in the posterior knee capsule^18^.

The increased concentration of type I collagen seen in capsular injuries causes a decrease in the ROM of the knee^19^. Thermosensitive hydrogels can absorb heat and provoke viscoelastic increased in this collagen. A temperature rise of 1 °C can have various effects on the human body, such as changes in nerve conduction velocity, enzyme activity and oxyhemoglobin release^20–23^. Tissue hypoxia results in tissue fibrosis and the production and release of algesic substances, causing pain, muscle spasm and joint contracture^24,25^. A temperature rise can improve oxygenated haemoglobin saturation^25^.

Physical therapies based on electrical or electromagnetic stimulation have been used in rehabilitation. Capacitive–resistive electric transfer (CRet) therapy has been used in physical rehabilitation to treat muscle, bone, ligament and tendon injuries^26–29^. CRet is a non-invasive electrothermal deep therapy, which is based on the application of electric currents within the radio frequency range of 300 kHz–1.2 MHz. This therapy can generate warming of deep muscle tissues and improve hemoglobin saturation^25^. The physiological effects of this type of physical therapy are generated by the application to the human body of an electromagnetic field with a frequency of about 0.5 MHz. The effects attributed to this technique include increased deep and superficial blood circulation, vasodilation, increased temperature, elimination of excess fluid and increased cell proliferation^30^. Some of these reactions, such as the increase in blood perfusion, are known to be linked to the temperature increase, but others, such as enhanced cell proliferation, seem to be mainly related to the passage of current^30^.

A previous article studied the changes in temperature with CRet vs hot pack. CRet was found to be more effective in treating musculoskeletal disorders than a hot pack. An important limitation that the authors discussed was that they used a non-invasive device to monitor deep tissue temperature instead of an invasive method using needles^25^.

The purpose of our in vitro study was to analyze the effects of different CRet protocols on the thermal behavior and transmission of electric current in superficial and deep knee tissues, by performing invasive temperature measurements on cadaveric specimens.

## Results

### Reliability

Reliability coefficients for all temperature locations were excellent. Standard errors of measurement and minimum detectable differences at 95% confidence interval were small (Table 1).

**Table 1:** Reliability of superficial, capsular and intra-articular temperature measurements.

Location	ICC	SEM	MDD
Superficial	0.94	0.07	0.19
Capsular	0..90	0.06	0.17
Intra-articular	0.99	0.05	0.15

*ICC* Intra-class correlation coefficient, *SEM* Standard error of measurement, *MDD* Minimum detectable difference.

### Baseline measurements

Descriptive outcomes of superficial, capsular and intra-articular temperature are shown in Table 2. The starting temperatures showed no statistically significant differences between treatment protocols in any of the positions (superficial *p* < 0.520; capsular *p* < 0.978; intra-articular *p* < 0.660). The current flow was stable, with averages of 0.104 A ± 0.06 (High Power Capacitive, HPC); 0.056 A ± 0.02 (Low Power Capacitive, LPC); 0.205 A ± 0.09 (High Power Resistive, HPR) and 0.092 A ± 0.5 (Low Power Resistive, LPR).

**Table 2:** Descriptive outcomes: temperature (°C).

	Baseline	1 min	2 min	3 min	4 min	5 min	5 min post-application
**Superficial**							
HPC	20.64 ± 1.25	27.71 ± 1.35	31.35 ± 1.54	34.35 ± 1.54	36.25 ± 1.80	37.95 ± 2.86	28.25 ± 2.14
LPC	20.47 ± 1.20	24.02 ± 1.20	26.21 ± 3.01	26.25 ± 1.55	27.13 ± 1.74	27.92 ± 1.83	22.73 ± 1.24
HPR	20.70 ± 0.96	26.60 ± 1.72	28.90 ± 2.42	31.05 ± 2.46	32.98 ± 2.74	34.27 ± 2.63	31.59 ± 3.12
LPR	19.95 ± 1.36	21.91 ± 1.35	22.32 ± 1.40	23.61 ± 3.56	22.91 ± 1.23	23.39 ± 1.27	22.02 ± 1.56
**Capsular**							
HPC	22.70 ± 1.55	24.73 ± 1.77	25.00 ± 1.88	25.46 ± 2.11	26.89 ± 2.27	26.29 ± 2.35	25.94 ± 1.89
LPC	22.67 ± 1.44	23.83 ± 1.38	24.14 ± 1.45	24.38 ± 1.41	24.55 ± 1.49	24.68 ± 1.54	24.77 ± 1.48
HPR	22.86 ± 1.38	28.12 ± 3.75	29.88 ± 4.18	31.35 ± 4.88	32.48 ± 5.28	34.22 ± 5.98	31.24 ± 4.00
LPR	22.79 ± 1.53	24.39 ± 1.81	24.87 ± 1.88	25.24 ± 2.00	25.54 ± 2.19	25.91 ± 2.26	25.47 ± 1.95
**Intra-articular**							
HPC	21.23 ± 2.65	23.38 ± 2.42	23.80 ± 2.75	24.10 ± 2.93	24.40 ± 3.08	24.73 ± 3.17	22.50 ± 1.65
LPC	22.15 ± 2.14	21.33 ± 1.79	21.49 ± 1.77	21.56 ± 1.79	21.67 ± 1.82	21.72 ± 1.84	21.19 ± 1.50
HPR	21.00 ± 2.48	24.55 ± 3.34	25.69 ± 4.12	26.51 ± 4.73	27.16 ± 5.05	28.14 ± 5.63	24.55 ± 3.71
LPR	21.88 ± 1.94	21.83 ± 1.34	22.10 ± 1.43	22.28 ± 1.48	22.45 ± 1.61	22.62 ± 1.67	21.45 ± 1.58

*HPC* high-power capacitive, *LPC* low-power capacitive, *HPR* high-power resistive, *LPR* low-power resistive.

All protocols showed a progressive increase in temperature at all depths, with subsequent decrease at 5 min post-application (*p* < 0.001 Friedman test), with the exception that the LPC and LPR treatments resulted in a slight decrease in temperature at 1 min in the intra-articular measurement and increased thereafter. LPR showed a slightly lower temperature than baseline at the 5 min post-application measurement.

### Superficial temperature

**Figure 1:**
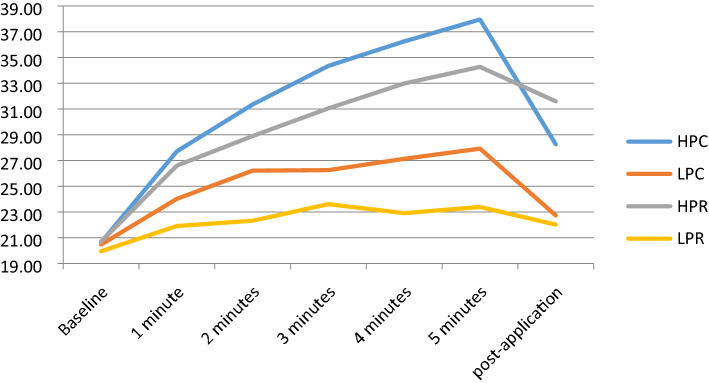
Superficial temperature. HPC high-power capacitive, LPC low-power capacitive, HPR high-power resistive, LPR low-power resistive.

The biggest increase in superficial temperature was found at the end of the treatment application in the HPC protocol: a superficial temperature of 37.95 °C, which represented an 84.2% increase from the starting temperature. However, this temperature decreased in the 5 min post-application to 28.25 °C, representing a 36.9% increase from baseline. The second highest superficial temperature was with HPR: 34.27 °C, representing a 65.4% increase from baseline. At 5-min post-treatment, the HPR protocol had the highest temperature, at 31.59 °C (52.4%), a decrease of 2.68 °C from the end of treatment, a milder decrease than the HPC that decreased 9.7 °C at the same measurement (Fig. 1).

The other two interventions (LPR and LPC) had lower percentage increases: 36.4% for LPC and 17.4% for LPR. These two protocols also had less of a temperature increase than the HPC and HPR at the post-application assessment, at 11.1% for LPC and 10.5% for LPR. There were statistically significant differences between the protocols for the difference between baseline and 5 min (of treatment) and between baseline and 5 min post-treatment, with the exception of the difference between LPC and LPR for baseline vs 5 min post-treatment (*p* < 0.579).

### Capsular temperature

**Figure 2:**
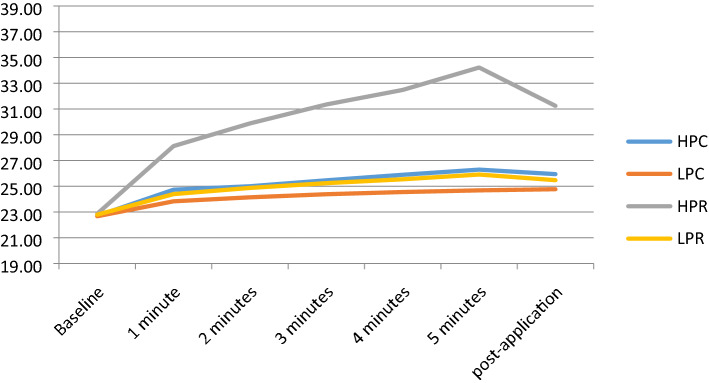
Capsular temperature. HPC high-power capacitive, LPC low-power capacitive, HPR high-power resistive, LPR low-power resistive.

In capsular temperature, HPR produced the biggest increase at 5 min: 34.22 °C, representing a 49.3% increase from baseline. This value decreased 2.98 °C in the 5 min post-treatment. In the other interventions, there was less of a temperature increase, the maximum being a 15.9% increase in the HPC protocol (Fig. 2).

Differences between HPC and LPC (*p* < 0.043), HPC and HPR (*p* < 0.001), LPC and HPR (*p* < 0.001) and between HPR and LPR (*p* < 0.001) were statistically significant. In the other interventions, no statistically significant difference was reached for baseline vs 5 min of treatment. A statistically significant difference was found between baseline and 5 min post-application, for HPR vs all other protocols (*p* < 0.001).

### Intra-articular temperature

**Figure 3:**
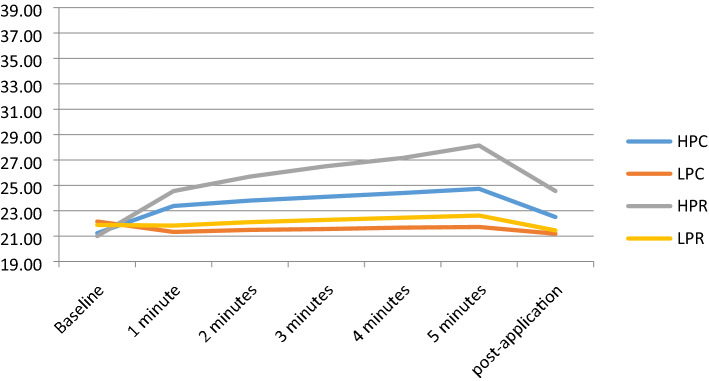
Intra-articular temperature. HPC high-power capacitive, LPC low-power capacitive, HPR high-power resistive, LPR low-power resistive.

The intra-articular temperature reached its highest value at 5 min with the application of HPR: 28.14 °C, representing a 34.3% increase from baseline. The second highest increase was 17.9 °C with HPC, and a decrease was even seen with LPC, of 0.43 °C, representing a 1.6% decrease. At 5 min post-application, temperature increased by 17.1% with HPR while with HPC it increased only 6.6%. LPC decreased by 4%, and LPR decreased by 1.8% (Fig. 3).

Differences were statistically significant between HPC and LPC (*p* < 0.007), LPC and HPR (*p* < 0.001), and HPR and LPR (*p* < 0.001) at 5 min of application. Differences between the other interventions did not reach statistical significance at this point. At 5 min post-application, there were statistically significant differences between HPC and LPC (*p* < 0.023), HPC and HPR (*p* < 0.019), LPC and HPR (*p* < 0.001) and HPR and LPR (*p* < 0.001).

## Discussion

As far as we know, this study is the first that evaluates the effects of CRet on temperature and current in deep structures in cadavers. The main findings divided by the protocol used are explained below.

At the end of treatment (5 min of treatment), Low-power capacitive obtained a 7.45 °C (35.40%) increase in superficial temperature, a 2.01 °C (8.96%) increase in capsule temperature and a 0.43 °C (1.62%) decrease in intra-articular temperature. This protocol slightly increases the superficial temperature without increasing the capsular or intra-articular temperature. However, despite the non-thermal effect, we observed a current flow (0.056 A ± 0.02), which has previously been shown to be related to cell proliferation in deep structures^30,31^. Recent literature reported that this type of application could be interesting in acute inflammatory intra-articular pathologies in which it is important to improve cell proliferation^30,31^ and tissue reconstruction without increasing the temperature, for example in ACL injury^4,6–10,25^, or even the treatment of scars^32^.

At 5 min of treatment, Low-power resistive obtained a 3.44 °C (17.48%) increase in superficial temperature, a 3.12 °C (13.69%) increase in capsule temperature and a 0.74 °C (3.81%) increase in intra-articular temperature. This type of application is similar to the LPC, however we can see that it has a lower superficial thermal effect and a non-thermal capsular and intra-articular effect with a greater current flow (0.092 A ± 0.5) than LPC^30,31,33^. This treatment may be useful in intra-articular pathologies, to increase cell proliferation^30,31^ with very little temperature change. It could be indicated for early intra-articular or capsular rehabilitation phases as reported in the literature^4,6–10,25^.

At 5 min, High-Power capacitive obtained a 17.31 °C (84.22%) increase in superficial temperature, a 3.59 °C (15.90%) increase in capsule temperature and a 3.50 °C (17.96%) increase in intra-articular temperature. With this protocol, we found an increase in temperature at all depths, especially the superficial level. In addition, we observed a high current flow (0.104 A ± 0.06), which is known to be associated with a cell proliferation effect^30,31^. This application could be interesting in more chronic phases in which the main objective is to improve the viscoelasticity of tissues, especially the capsule and ligaments, since, as reported in the literature, these structures are directly related to limitation of ROM after prolonged immobilization or chronic pathologies^17,18,25^.

At 5 min, High-Power resistive obtained a 13.57 °C (65.42%) increase in superficial temperature, an 11.36 °C (49.13%) increase in capsule temperature and a 7.14 °C (34.26%) increase in intra-articular temperature. This setting achieved the greatest increase in temperature in the capsule and intra-articular structures. It also recorded the highest current flow (0.205 A ± 0.09), which has been associated with a cell proliferation effect^30,31,33^. This application has a greater effect on deep structures than HPC and could be combined with it. The thermal and current effect may generate mechanical effects on the viscoelastic properties of the structures, which are associated with pain and loss of ROM^17,18,25^.

## Conclusion

The low-power treatments demonstrated minimal capsular and intra-capsular thermal effects, but an electric current flow was observed. These low-power CRet protocols could be indicated for treatments in inflammatory pathologies in which a temperature increase is not of interest.

High-power treatments achieved a greater increase in capsular and intra-articular temperature and a higher current flow than low-power treatments. HPR gave the highest capsular and intra-capsular temperatures. It could be indicated for treatment in chronic pathologies in which it is desirable to increase the deep temperature to generate viscoelastic changes in deep structures.

Low- and high-power capacitive treatments achieve a greater increase in superficial temperature.

More studies are needed in living subjects to support these findings.

## Limitations

The results of this study on cadavers may differ from studies on living subjects. Functional thermoregulation mechanism was not possible in our sample and it is probable that tissues from living subjects may experiment less increase of temperature as circulating blood would dissipate the heat throughout adjacent body areas. This thermoregulation and the patient feedback also ease avoiding an unwanted hyperthermia and a potential burning of the skin^25^. In addition, despite being fresh corpses, it is very likely that the capsular and muscular properties were not the exactly the same as those of living subjects. Nonetheless, this in vitro study with cryopreserved cadavers allowed to measure the tissue temperature in the deep tissues of the knee joint and to make hypothesis about what happens when the CRet therapies are applied in living real patients.

## Methods

### Study design

This was a cross-sectional study designed to determine the effect of resistive energy/electrical capacitive transfer of the T-Plus Wintecare device on temperature in the intra-articular, capsular and superficial region of the knee in cadaveric specimens. The body donor program of the Faculty of Medicine and Health Science of Universitat Internacional de Catalunya (UIC) provided all specimens. Permission for the use of the cadavers in the study was obtained from the Anatomy Lab of this university. A local committee (CER, Comite d’Ètica de Recerca, UIC) approved the study.

### Cadaveric specimens

The study sample included 5 fresh frozen cadavers, 4 males and 1 female (10 legs). The mean age at the time of death was 69.80 ± 6.04 years. The cadavers were stored at 3 °C and brought to room temperature before testing. None of the cadavers used for this study had evidence of trauma or surgical scars on the limbs.

### Intervention

To simulate the conditions of a real CRet clinical application and to understand the consequent temperature change and the passage of electric current, we used a T-Plus model with similar power limits as applied during treatments with real patients. This was based on the power level, which is easily identifiable and controllable by the therapist during therapy, and the watts (the absorbed power) shown by the device during the application^34^.

The power range of a very large T-Plus device ranges from 1 to 300 watts in resistive mode and from 1 to 450 VA in capacitive mode.

Two thresholds were identified for *high power* and *low power,* based on the real powers that the therapist typically applies when he/she wants to induce a thermal or non-thermal reaction, respectively. CRet therapy provides two different treatment modes: capacitive and resistive. Both treatment modes induce different tissue responses depending on the resistance of the treated tissue^34^. Capacitive mode is provided with an insulating ceramic layer and the energetic transmission generates heat in superficial tissue layers, with a selective action in tissues with low-impedance (water rich)^34^. Resistive mode has no insulating ceramic layer, the radiofrequency energy passes directly through the body in the direction of the inactive electrode, generating heat in the deeper and more resistant tissues (with less water content)^34^. Based on this, *high power* was defined as application at **130 VA in capacitive mode** (HPC) **and 100 watts in resistive mode** (HPR)*,* while *low power* was defined as application at **50 VA in capacitive mode** (LPC) **and 20 w in resistive mode** (LPR). Compared to the average real-life use, these low-power thresholds (20 w; 50 VA) respect the limit of 0.3 A, while the high-power thresholds (100 watts; 130 VA) will be above 0.3 A and therefore expected to generate thermal effects.

**Figure 4:**
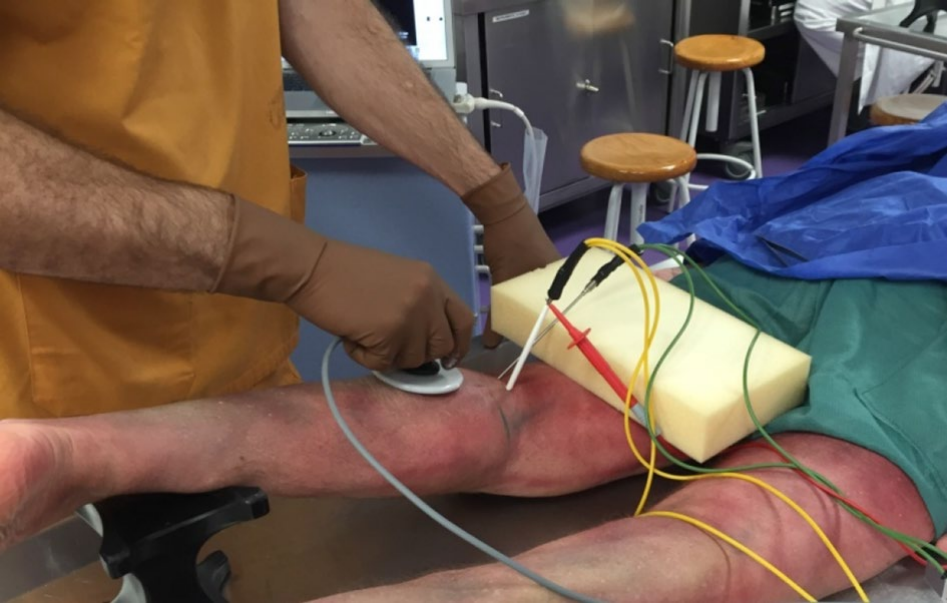
Intervention with T-Plus Wintecare.

The 4 interventions (capacitive and resistive mode; low- and high-power) were performed for 5 min each, by a physiotherapist with experience in the use of T-Plus. The time of application was established in a previous study, similar to the one carried out^34^. Dynamic movements similar to those used with real patients were performed with constant pressure to the posterior region of the knee (Fig. 4). For the resistive applications conductive cream was applied during the treatment. For capacitive applications no cream was applied during treatment.

### Experimental procedures

Each cadaver was placed in the prone position. The hips were positioned in a neutral rotation, the knee in 30° of flexion, and the ankle joint position was maintained using a thermoplastic splint.

The order of the 4 treatment protocols was previously randomized, as was as the specimen (leg). For the randomization process, an external evaluator generated a random assignment list before the study begins with a computer program (www.random.org) that generated a list of sequential numbers. The temperature generated in the cadaver was allowed to return to baseline before the next treatment was applied.

**Figure 5:**
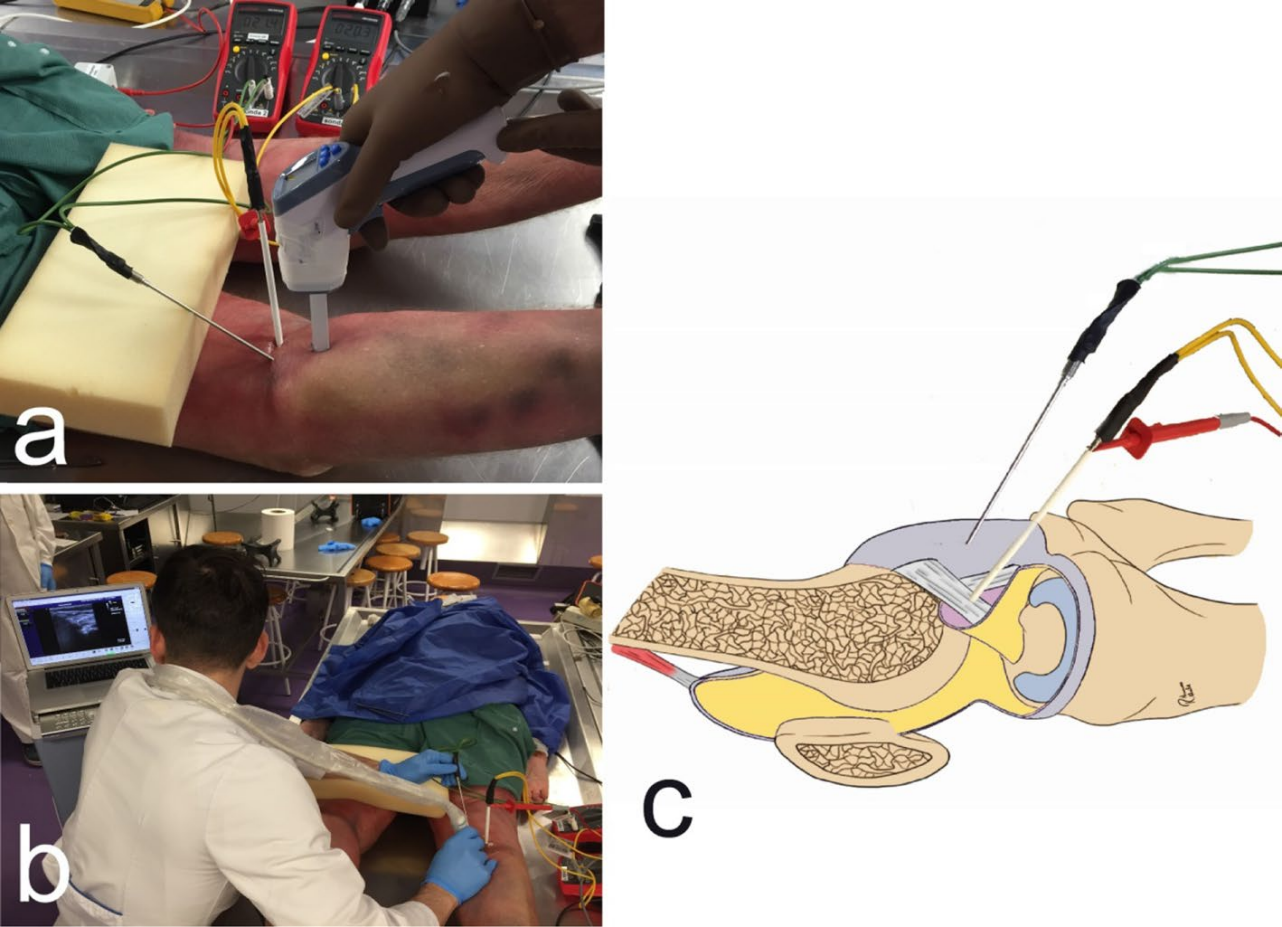
(**a**) Temperature measurement with digital thermometer, (**b**) Thermocouple placement under ultrasound guidance, (**c**) Thermocouple placement.

All instrumentation received a calibration certificate prior to this study. Thermocouples “Hart Scientific PT25 5628–15” were used to monitor the intra-articular and capsular temperature ( °C) of the knee. A digital thermometer “Thermocomed” was used to measure the superficial temperature (Fig. 5a). The thermocouples were placed under ultrasound guidance “US Aloka Prosound C3 15.4”, with a high-frequency linear transducer (USTTL01, 12L5), by an expert in the use of the instrument (Fig. 5b)^34^. The deeper thermocouple was placed intra-articularly and the other in contact with the posterior tibiofemoral capsule (Fig. 5c).

The return electrode of the T-Plus was placed on the abdomen of the specimen and the treatment was carried out with the movable electrode of the T-Plus on the back of the knee for 5 min. The superficial, capsular and intra-articular temperatures were measured. These measurements were recorded at 1-min intervals for 5 min, then at 5 min after the end of each treatment. Prior to the treatment, impedance was always measured (Multimeter Fluke 8846A) to ensure that the values marked by the T-Plus Wintecare device were correct. In addition, the current flow of each application was calculated (average voltage divided by the initial impedance)^34^.

### Statistical analysis

Analyses were performed using SPSS Statistics version 22.0.

The intra-class correlation coefficient (ICC) at a 95% confidence interval (CI), the standard error of measurement (SEM) and the minimum detectable difference (MDD) were calculated for the superficial, capsule and intra-articular temperature measurements. The following interpretation of ICCs was considered (0.00 to 0.25 = little to no relationship, 0.26 to 0.50 = fair degree of relationship, 0.51 to 0.75 = moderate to good relationship, and 0.76 to 1.00 = good to excellent relationship)^35^.

The normality of the distribution was assessed with the Shapiro–Wilk test (*p* > 0.05). Mean and standard deviation of the superficial, intra-articular and capsular temperature were calculated.

The percentages of temperature change respect to baseline temperature were calculated.

The Friedman test and Wilcoxon signed-rank test were used for intra-treatment differences. The Kruskal–Wallis test and Mann–Whitney U test were performed for between-treatment comparisons. A *p* value < 0.05 was considered statistically significant^34^.

### Ethics approval

The Comité d´Ètica de Recerca from Universitat Internacional de Catalunya approved the study (CBAS 2019-07). The investigation conformed with the principles outlined in the Declaration of Helsinki. The informed consent from "body donors" was obtained before the death and any personal data was hidden.

## Data Availability

The datasets used and/or analysed during the current study are available from the corresponding author on reasonable request.

## Abbreviations

ROM: Range of motion
ACL: Anterior cruciate ligament
CRet: Capacitive–resistive electric transfer
HPC: High-power capacitive
LPC: Low-power capacitive
HPR: High-power resistive
LPR: Low-power resistive
